# Integrating musculoskeletal simulation and machine learning: a hybrid approach for personalized ankle-foot exoskeleton assistance strategies

**DOI:** 10.3389/fbioe.2024.1442606

**Published:** 2024-08-06

**Authors:** Xianyu Zhang, Shihao Li, Zhenzhi Ying, Liming Shu, Naohiko Sugita

**Affiliations:** ^1^ Department of Mechanical Engineering, The University of Tokyo, Tokyo, Japan; ^2^ School of Mechanical Engineering, Dalian University of Technology, Dalian, China

**Keywords:** lower limb exoskeletons, musculoskeletal simulation, machine learning, human-robot interaction, walking augmentation

## Abstract

**Introduction:** Lower limb exoskeletons have shown considerable potential in assisting human walking, particularly by reducing metabolic cost (MC), leading to a surge of interest in this field in recent years. However, owing to significant individual differences and the uncertainty of movements, challenges still exist in the personalized design and control of exoskeletons in human-robot interactions.

**Methods:** In this study, we propose a hybrid data-driven approach that integrates musculoskeletal simulation with machine learning technology to customize personalized assistance strategies efficiently and adaptively for ankle-foot exoskeletons. First, optimal assistance strategies that can theoretically minimize MC, were derived from forward muscle-driven simulations on an open-source dataset. Then, a neural network was utilized to explore the relationships among different individuals, movements, and optimal strategies, thus developing a predictive model.

**Results:** With respect to transfer learning, our approach exhibited effectiveness and adaptability when faced with new individuals and movements. The simulation results further indicated that our approach successfully reduced the MC of calf muscles by approximately 20% compared to normal walking conditions.

**Discussion:** This hybrid approach offers an alternative for personalizing assistance strategy that may further guide exoskeleton design.

## 1 Introduction

Ankle-foot exoskeletons (AFEs) have shown considerable potential in enhancing human mobility (Lee et al., 2020). In recent decades, a variety of AFEs have been introduced (Collins et al., 2015; Mooney and Herr, 2016; Galle et al., 2017; Zhang et al., 2017; Nuckols and Sawicki, 2020; Slade et al., 2022), each aiming to optimize walking efficiency--a key factor that reduces fatigue, increases comfort and extends usage time (Sawicki et al., 2020). Despite these efforts, achieving significant improvements in the walking economy has proven challenging, with only modest advancements realized relative to the substantial benefits anticipated. For example, Collins developed a passive AFE that offloads muscle forces by incorporating a spring parallel to calf muscles, achieving a metabolic cost (MC) reduction of 0.21 w/kg compared to normal walking (Collins et al., 2015). Mooney introduced an active AFE powered by a winch actuator that indirectly supports ankle plantar flexion (PF), reducing the MC by 0.43 w/kg (Mooney and Herr, 2016). Similarly, Galle designed a tethered AFE that partially substitutes muscle effort with a pneumatic muscle, reducing the MC by 0.44 w/kg (Galle et al., 2017).

Personalizing assistance is essential for AFEs to effectively reduce MC. However, developing a personalized assistance (PA) strategy adaptable to users and movements remains challenging (Young and Ferris, 2016). Traditional methods typically adjust predefined torque profiles and apply compliance-based impedance/admittance control. For instance, Chinimilli et al. (2019) introduced an impedance-tuning method for knee exoskeletons based on activity and gait phases. However, such methods may not sufficiently lower the MC or are limited to specific movements. Recently, human-in-the-loop optimization methods have emerged, which utilize physiological signals to assess movement performance and refine assistance strategies. Slade et al. (2022); Kantharaju et al. (2022) utilized respiratory data to generate PA strategies for walking and squatting, respectively. However, these methods are time-intensive and typically require several minutes to achieve convergence in optimizing these strategies. They also presuppose a steady-state movement process, which is rarely consistent in real-world scenarios. Additionally, the practicality of wearing a respiratory mask during daily activities is questionable, posing another hurdle to the widespread adoption of such methods.

Musculoskeletal (MS) simulation is a powerful tool for investigating human motion (Seth et al., 2018) and human-robot interactions (Sartori et al., 2018; Durandau et al., 2022). It enables the analysis of physiological signals that are otherwise challenging to measure directly. For example, Durandau et al. (2017) utilized an electromyography (EMG) driven MS model to infer muscle forces from EMG signals. Peternel et al. (2019) employed MS simulation to estimate muscle forces and fatigue levels. MS simulation provides a cost-effective alternative to experiments for exploring the impact of various types of assistance on movement and guiding exoskeleton design. Notably, Uchida et al. (2016); Dembia et al. (2017) employed MS models coupled with massless actuators to investigate optimal assistance for running and weight-bearing walking, respectively.

Despite significant achievements, MS simulation faces limitations in terms of time consumption and adaptability. These challenges stem from the necessity to rerun simulations for minor variations among individuals or movements, an inability to utilize historical data for future movement predictions, and an overreliance on extensive experimental data and expertise. To address these limitations, recent studies have explored integrating MS simulation with machine learning (ML) techniques. For instance, Burton et al. (Burton et al., 2021) employed ML to swiftly estimate muscle and joint forces following initial calculations using MS simulation. Similarly, Sharma et al. (Sharma et al., 2022) utilized MS simulation to gather biomechanical data on upper extremity movements, which they then processed using ML algorithms for quick prediction. Other related research includes studies focused on predicting ground reaction forces (Wouda et al., 2018), implant pressure distributions (Ardestani et al., 2015), and the deformability of joint contacts (Eskinazi and Fregly, 2015). These hybrid methods demonstrate advantages over traditional MS simulation. However, the application of this approach to exoskeleton design and human-robot interactions remains relatively unexplored. Motivated by these advances, our research further investigates the dynamic interplay between exoskeleton assistance and biomechanics, to uncover more effective patterns of human-robot interaction.

**FIGURE 1:**
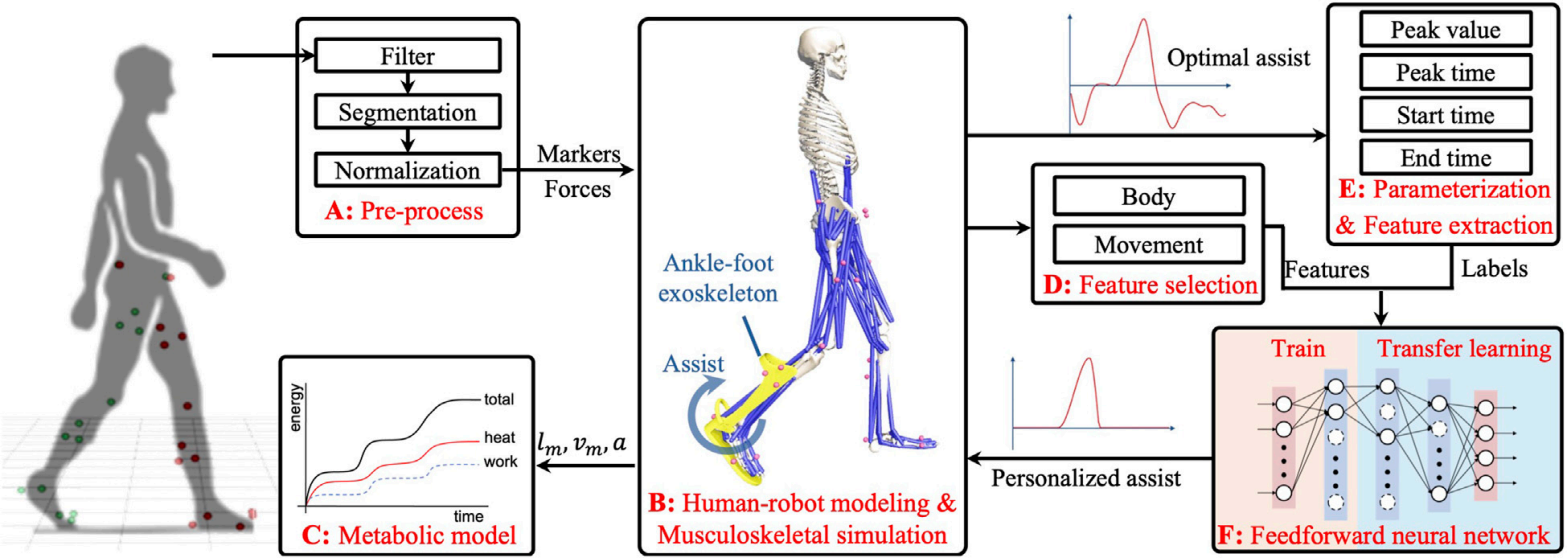
Schematic outlining the framework of the proposed method. **(A)**: Preprocessing of raw gait data. **(B)**: Human-robot modeling and forward muscle-driven simulation for optimal assistance. **(C)**: Metabolic cost calculation model. **(D,E)**: Feature extraction from the optimal strategies and feature selection. **(F)**: Training and transfer learning process of an FNN for generating PA strategies.

Personalizing assistance faces two primary challenges: variability in movements and significant individual differences. This study proposes a novel hybrid approach that combines MS simulation with ML to tackle the inherent challenges, as illustrated in Figure 1. This method uses MS simulation initially to identify optimal assistance (OA) strategies tailored for individuals across different walking scenarios. Subsequently, a feedforward neural network (FNN) model is employed to detect patterns and relationships between individual characteristics, movements, and OA features. Leveraging transfer learning, the trained model adapts to new users and movements. The main advantages of the proposed method include the following: 1) Utilizing the strengths of MS simulation in movement analysis and assistance customization, it quantifies and optimizes user performance, ensuring the effectiveness of PA strategies in reducing MC. 2) Integrating ML capabilities, facilitates the generation of PA strategies for new users and movements, addressing traditional limitations such as time consumption and inflexibility. Through simulation validation, our results show that the PA strategy can significantly reduce MC by approximately 20% compared to normal walking, demonstrating its potential efficacy and applicability.

## 2 Materials and methods

Our objective is to develop a comprehensive and effective approach for the automated generation of PA strategies that are applicable to diverse individuals and movements. We utilized OpenSim (Seth et al., 2011) for MS simulation. The gait data, comprising marker trajectories and ground reaction forces, were derived from the Camargo dataset (Camargo et al., 2021). What follows is an in-depth exposition of our approach.

### 2.1 Forward muscle-driven simulation for optimal assistance

We conducted forward muscle-driven simulations to identify OA strategies that minimize MC. These simulations utilized a generic MS model with 23 degrees of freedom named Gait2392 (Delp et al., 1990). Subject-specific models were customized by scaling this generic model. To enhance the realism of human-robot interactions and ensure universality, we designed an AFE using SolidWorks. This AFE comprises two main components: a cuff attached to the calf and a sole secured to the foot, interconnected by a hinge at the ankle joint to enable rotational movement. To prevent the AFE from imposing constraints on normal ankle joint movement, we fixed the cuff part within the tibia coordinate frame, and the sole part within the calcaneus coordinate frame of the MS model. To address the potential misalignment issues, we meticulously adjusted the relative positions of the two parts within their respective coordinate frames during the initial standing position for each participant. This adjustment ensured the alignment of the exoskeleton’s rotational axis with that of the human ankle joint as closely as possible. Assistance is simulated by applying an external torque at this hinge, effectively mimicking the support from AFEs. Additionally, we accounted for the extra mass of the AFE by assigning 1.5 kg to the cuff and 0.5 kg to the sole.

**FIGURE 2:**
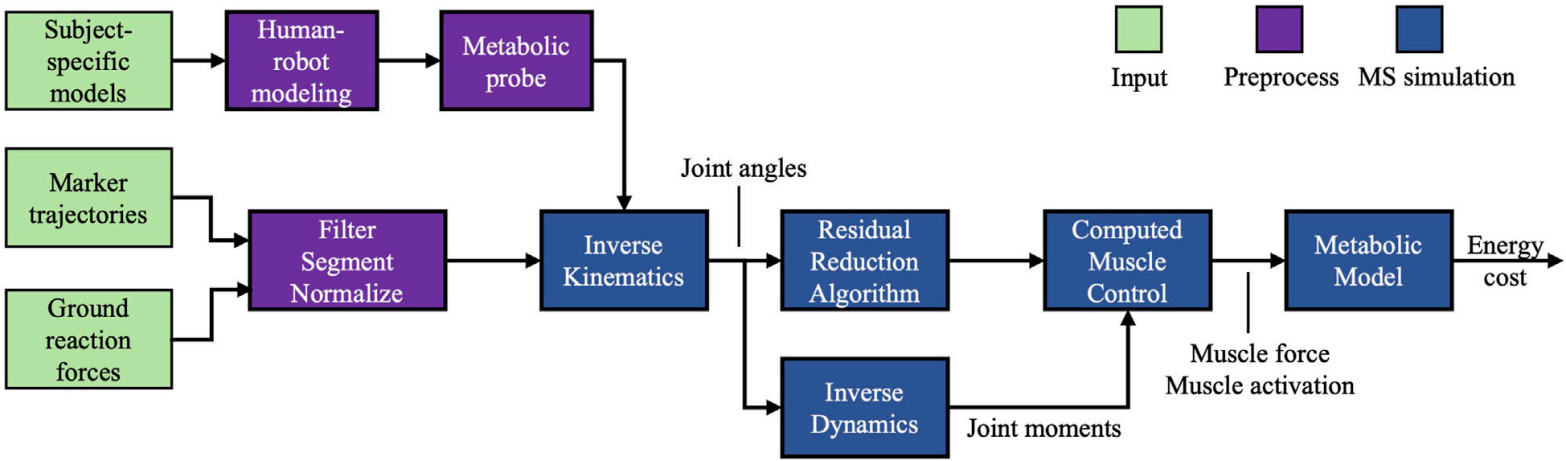
Flowchart of the simulation. Input: subject-specific model and experimental gait data. Preprocess: experimental data were processed, the human-AFE models were coupled, and metabolic probes were added. Simulation: forward muscle-driven simulation with metabolic cost calculation.

Inverse kinematics and inverse dynamics were executed to determine joint angles, and the net biological moments, respectively. To improve the consistency between the simulation results and the experimental results, we employed the residual reduction algorithm. Subsequently, we employed the computed muscle control (CMC) algorithm (Thelen and Anderson, 2006), an algorithm for forward muscle-driven simulation, to compute a series of muscle activations that minimize the overall instantaneous MC within the defined kinematic and kinetic constraints. Figure 2 depicts the flowchart of our simulation.

In our simulations, we employed the Hill-type muscle model (Zajac, 1989) to elucidate the mechanism of muscle force $F_{muscle}$ with the following mathematical model (Eq. 1):

$$F_{muscle,i}=\phi \left(l_{i},v_{i},a_{i},P_{i}\right)$$ (1)

where $l_{i}$ represents the normalized length of muscle *i*; $v_{i}$ denotes the muscle’s contraction velocity; $a_{i}$ is the activation level, $a_{i}\in \left[0.02,1\right]$ ; $P_{i}$ includes muscle-specific parameters such as the maximum isometric force; and ϕ functions as a mapping relation. To optimize the CMC problem, we integrated residual and reserve actuators as external torque providers at each joint. These actuators are crucial for providing additional torque when muscle-generated forces are insufficient, particularly in scenarios where joints experience higher-than-expected loads. The CMC computes a set of muscle activations that minimize the following instantaneous cost function:

$$\min J=\sum_{i=1}^{N_{muscles}}a_{i}^{2}+\sum_{i=1}^{N_{reserves}}{\left(\frac{\tau_{j}}{w_{j}}\right)}^{2}+\sum_{i=1}^{N_{residuals}}{\left(\frac{\tau_{k}}{w_{k}}\right)}^{2}$$ (2)

where $N_{muscles}$ , $N_{reserves}$ and $N_{residuals}$ denote the number of muscle-tendons, reserve, and residual actuators, respectively; $\tau_{j}$ and $\tau_{k}$ are the instantaneous output torques of the reserve and residual actuators; and $w_{j}$ and $w_{k}$ are constants that signify the penalty factors, with a higher $\left(\tau_{j}/w_{j}\right)$ ratio incurring a greater penalty. In Equation 2, the first term represents the square of muscle activation, which is also proportional to muscle MC. The subsequent terms function as penalty terms for the utilization of reserve and residual actuators. These actuators are designed to remain inactive unless necessary, being activated only when muscle-generated torques alone are inadequate. Consequently, the weighting factors w are typically set to 1. Additionally, the following constraint (Eq. 3) must be satisfied for each joint *m*:

$$\tau_{net,m}=\sum_{i=1}^{N_{muscles}}r_{i,m}\times F_{muscle,i}+\sum_{j=1}^{N_{reserves}}\tau_{j}+\sum_{k=1}^{N_{residuals}}\tau_{k}$$ (3)

where $\tau_{net,m}$ is the net biological moment of joint *m*; $r_{i,m}$ is the moment arm of muscle *i* for joint *m*.

To simulate AFE assistance and deriving the OA strategy, we adopted the method proposed by Uchida (Uchida et al., 2016). We introduced an ideal actuator at the right ankle joint, to satisfy the net moment demands while simultaneously reducing the effort exerted by the leg muscles. This actuator, an evolved version of the reserve actuator, boasts the ability to apply torques of any magnitude and direction directly to the ankle joint. Different from the reserve actuator, we adjusted its parameters to encourage the optimizer could employ it freely. Specifically, its penalty factor $w_{ideal}$ , was increased from 1 to $10^{6}$ , implying that even a substantial torque it provides incurs a negligible penalty. After obtaining the muscle activations, we utilized the MC model proposed by Umberger (2010) to calculate the instantaneous metabolic rate, $\dot{E_{i}}$ , for each muscle *i*. Detailed calculations of $\dot{E_{i}}$ are available in the referenced publication. We then aggregated the outcomes across all muscles and conducted a time-based integration. The resulting value was then divided by the gait duration T and normalized by the subject’s mass M to yield the total metabolic power (in W/kg, Eq. 4):

$$E=\frac{1}{MT}\int_{0}^{T}\left(\sum_{i=1}^{Nmuscles}\dot{E_{i}}\left(t\right)\right)dt$$ (4)

This study utilized a subset of the Camargo dataset to perform MS simulations. The selected data comprised 22 healthy subjects (9 females and 13 males, age: 21.6±3.58 years, mass: 68.5±11.33 kg, height: 1.71±0.07 m, mean ± SD) walking on a treadmill at 14 distinct velocities (ranging from 0.5 to 1.8 m/s in 0.1 m/s increments). The raw data captured 30 continuous seconds of each subject’s movement at every speed level. We segmented the raw data into gait cycles based on the right heel strike event and normalized each cycle from 0% to 100% in 1% increments. The number of gait cycles recorded for each subject varied with speed, ranging from approximately 20 cycles at 0.5 m/s to 40 cycles at 1.8 m/s. After omitting any incomplete gait cycles, we used a total of 8,935 cycles for simulation. On average, each subject contributed approximately 400 cycles to the simulation. The Camargo dataset provides a subject-specific model for each participant. Additionally, to improve the mechanical reliability of each model, dynamic parameters were adjusted before the formal simulations to reduce discrepancies between simulated muscle excitations and EMG data.

### 2.2 Feedforward neural network for personalized assistance

To explore the potential relationships between individuals, their movements, and the derived OA strategies, we deployed an FNN model. We anticipated that this model, when fed with specific body features and movement characteristics, could swiftly devise PA strategies that are effective in reducing MC.

#### 2.2.1 Selection of body features and movement characteristics

Previous studies (Chehab et al., 2017; Embry et al., 2018; Reznick et al., 2021) have emphasized that physiological attributes such as age, sex, height, mass, and body mass index significantly influence gait patterns. In this study, given that young adults had minimal age variance, age was not considered a suitable feature and was excluded from the body features. Additionally, after conducting a feature analysis, we observed a pronounced correlation between the dimensions of body segments--specifically the lengths of the thigh, calf, and foot, as well as the dimensions of the torso and pelvis--and the assistance strategy. Thus, these features were included as critical input body features. The various movements primarily manifest as walking speed. Furthermore, we also identified disparities in stride length, cadence, and maximum heel clearance. As a result, these movement characteristics were incorporated as additional features in our analysis.

#### 2.2.2 Parameterization and feature extraction of the optimal assistance

**FIGURE 3:**
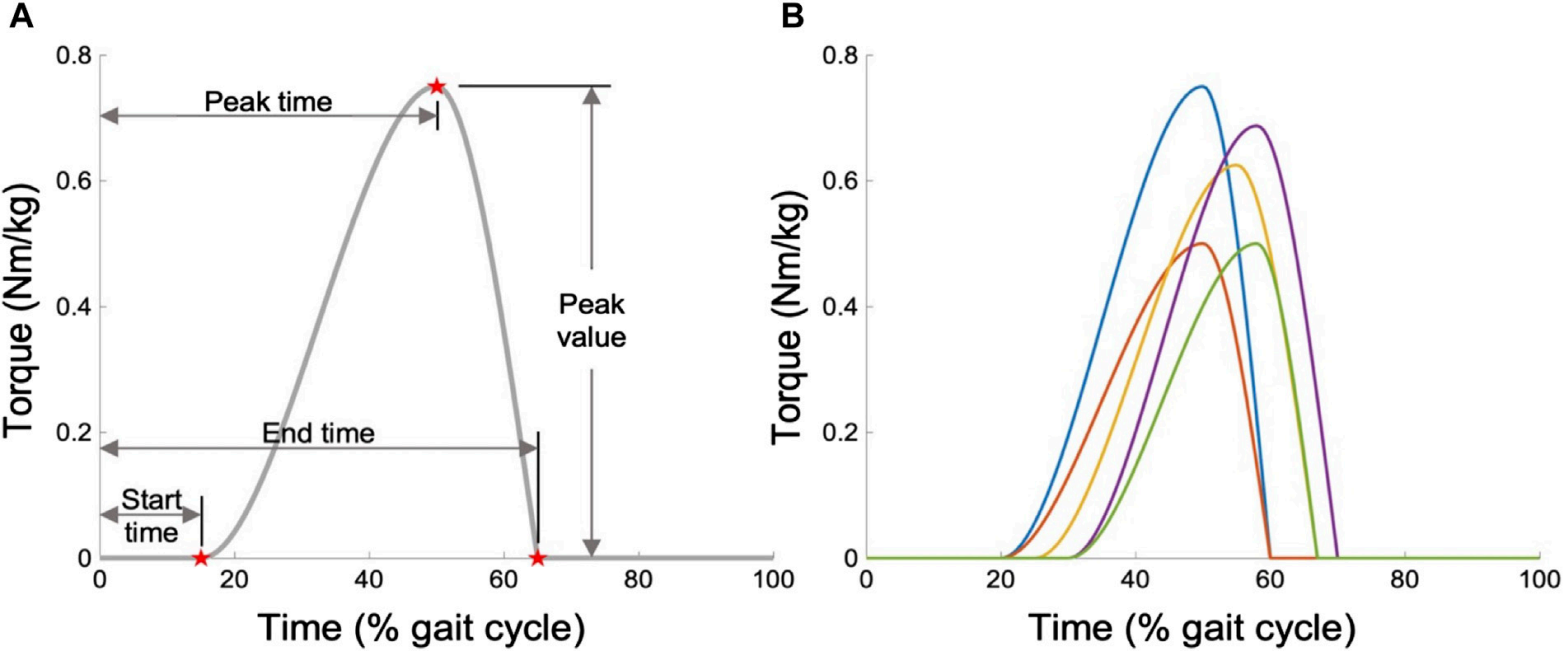
Parameterization of the OA strategy. **(A)** Generic torque profile template, defined as a function of time through four features. **(B)** Examples of possible profiles.

During the modeling process, we enabled the AFE to support both ankle PF and dorsiflexion (DF) movements, imposing no constraints on the optimization of OA strategies. However, assisting in both directions generally requires sophisticated hardware capabilities beyond what is typically available in most existing AFE designs, which typically support PF only (Collins et al., 2015; Mooney and Herr, 2016; Galle et al., 2017; Zhang et al., 2017; Nuckols and Sawicki, 2020; Slade et al., 2022). Moreover, studies like Dembia et al. (2017) suggest that focusing exclusively on PF assistance is a more cost-effective method for reducing MC than offering dual-direction support. Consequently, after optimization, we refined our OA strategy to exclusively assist in the PF direction, while eliminating assistance in the DF direction. To standardize the expression of all OA strategies, we adopted a piecewise cubic function as a universal template, which is depicted in Figure 3A. This template is defined by four key parameters: peak value, peak time, start time, and end time. The function of the assistance profile is mathematically articulated as follows (Eq. 5):

$$f\left(t\right)=\left\{\begin{array}{c} f_{1}\left(t\right)=a_{1}t^{3}+b_{1}t^{2}+c_{1}t+d_{1},t_{1}\leq t\leq t_{2}\\ {\;f}_{2}\left(t\right)=a_{2}t^{3}+b_{2}t^{2}+c_{2}t+d_{2},t_{2}\leq t\leq t_{3}\\ 0,otherwise \end{array}\right.$$ (5)

while satisfying the following constraints (Eq. 6):

$$\begin{array}{c} f_{1}\left(t_{1}\right)=0,f_{1}\left(t_{2}\right)=\tau_{peak},f_{2}\left(t_{2}\right)=\tau_{peak},f_{2}\left(t_{3}\right)=0\\ f_{1}^{\prime }\left(t_{1}\right)=0,f_{1}^{\prime }\left(t_{2}\right)=0,f_{2}^{\prime }\left(t_{2}\right)=0,f_{2}^{\prime }\left(t_{3}\right)=0 \end{array}$$ (6)

where $t_{1}$ , $t_{2}$ , and $t_{3}$ denote the start, peak, and end times, respectively. By adjusting the generic template, we can derive distinct profiles tailored to different individuals and movements. The generic template yields multiple possible profiles, as depicted in Figure 3B. To distill the profile features from the simulation outcomes, the OA strategy was first normalized over the time axis from 0% to 100% in 1% increments. Subsequently, a zero-lag 6th-order Butterworth low-pass filter with a cutoff frequency of 10 Hz was applied to mitigate noise. Then the filtered profile underwent full-wave rectification. Finally, we extracted the four features of each profile via the template matching method.

#### 2.2.3 Construction and training of the feedforward neural network model

To generate PA strategies, we employed a standard fully connected FNN as a substitute for MS simulation. A grid search was conducted to optimize the hyperparameters of this FNN. The network architecture comprises an input layer with 13 nodes, each representing specific body features and movement characteristics as detailed in Section 2.2.1. This is followed by three hidden layers with 32, 24, and 12 nodes, respectively. It concludes with an output layer of four nodes, with each corresponding to one of the profile features as explained in Section 2.2.2. The rectified linear unit served as the activation function. We utilized the L1 norm as the loss function to minimize the sum of the absolute differences between actual and predicted values for all four profile features, formulated as (Eq. 7):

$$L=\sum_{i=1}^{N}\sum_{j=1}^{4}\left|y_{ij}-{\hat{y}}_{ij}\right|$$ (7)

where $y_{ij}$ represents the actual value and ${\hat{y}}_{ij}$ denotes the predicted value for the *jth* profile feature of the *ith* sample, and *N* is the number of samples. This loss function was chosen for its robustness to outliers, a quality that is crucial for handling the high variability inherent in human gait data. The FNN was developed using PyTorch with the Adam optimizer. The network was trained over 200 epochs, incorporating early stopping mechanisms to prevent overfitting. The initial learning rate was set at 0.001 and was dynamically adjusted based on the loss rate. We set the batch size at 64 and implemented a dropout rate of 0.1 and L2 regularization ( λ=0.001 ) to enhance the model’s generalization capabilities and mitigate overfitting.

The training dataset comprised 20 subjects randomly chosen from a pool of 22 subjects. Each subject contributed 300 samples randomly selected from approximately 400 available samples. These selected samples spanned 14 different speeds, with at least 10 samples per speed, yielding a total of 6,000 samples. We utilized cross-validation, allocating 80% of the shuffled training dataset for training and the remaining 20% for validation. The remaining two subjects, not included in the training group, were used as the test dataset; similarly, 300 samples were randomly selected for each subject, totaling 600 samples. We observed a notable improvement in prediction performance through the application of transfer learning using partial data from the test subjects before actual testing. This approach not only improves the network’s adaptability to new individuals but also demonstrates practical viability in real-world settings. Specifically, we used gait data from the two test subjects at extreme speeds of 0.5 m/s and 1.8 m/s for transfer learning, and the remaining data were used for evaluation.

### 2.3 Evaluation of simulation results and the FNN model

In this study, approximately ten thousand MS simulations were performed. To verify the reliability of our simulations, we evaluated various parameters, including positional errors and the magnitudes of residual/reserve actuators. For instance, a comparison of the inverse kinematic results with experimental marker trajectories indicated that both the maximum and the root mean square positional errors were maintained below 4° (2 cm). This level of accuracy signifies a high consistency between the simulation results and experiments. Furthermore, the peak values of the reserve/residual actuators, which were less than 25 (60) Nm, confirmed the reliability of our dynamic results. All these parameters fell within the best practice thresholds recommended by OpenSim (Opensim Documentation, 2024), confirming the validity of our simulation results.

To assess the performance of the FNN, we analyzed its prediction accuracy both before and after applying transfer learning using the same test dataset. Additionally, we assessed the effectiveness of the PA strategy in reducing MC across three distinct walking scenarios: slow (1 m/s), normal (1.3 m/s), and fast (1.6 m/s). Five gait cycles per test subject were randomly selected at each speed for analysis. Utilizing the MC model, we compared the impacts of different assistance strategies on the MC.

## 3 Results

We performed a qualitative analysis of the kinematic and dynamic disparities among individuals across various movements. We analyzed the variations in OA strategies under different conditions and the trends of four assistance features concerning speed. The prediction accuracy of the FNN was quantitatively assessed. Additionally, we thoroughly compared the effects of different types of assistance on both overall and muscle-specific MC at various speeds. All the results were derived from simulations.

### 3.1 Gait analysis from kinematic and kinetic results

**FIGURE 4:**
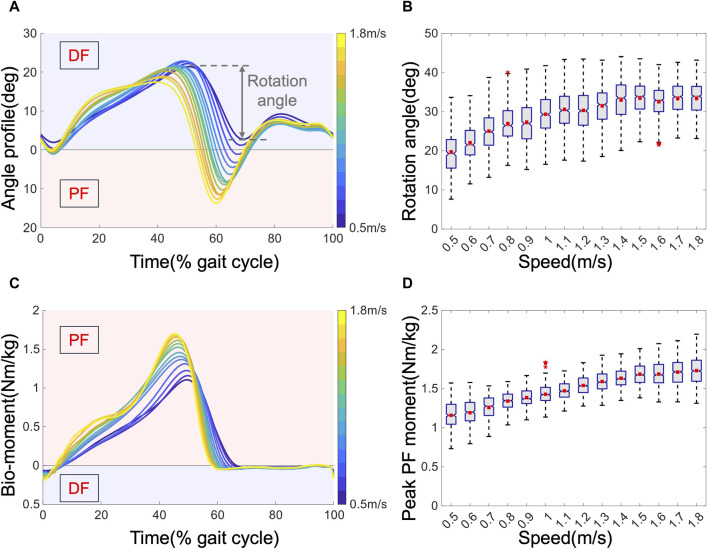
Kinematic and kinetic results of the ankle joint. **(A)**. Average angle profiles across all subjects. **(B)**. Rotation angle versus speed. Boxes extend from the lower quartile to the upper quartile, with notches at the median and squares at the mean. The whisker length = 1.5 interquartile range, and outliers are marked with asterisks. **(C)**. The average biological torque was normalized by mass. **(D)** Peak PF moment.

The joint angles and biological moments at the ankle joint are depicted in Figure 4. The kinematic results are presented in Figures 4A, B. The variation in joint kinematics with increasing speed indicates that human walking patterns are not fixed, which is consistent with previous results (Winter 2009). Figure 4A displays the average angle profiles for all subjects within one gait cycle. Notably, as the speed increases, the maximum DF angle decreases from approximately 25 to 15°, while the maximum PF angle increases from approximately 0–15°, accompanied by an earlier peak PF occurrence from approximately 70%–60% of the gait cycle. Figure 4B details the relationship between the rotation angle and speed. The rotation angle, defined as the ankle’s range of motion during the push-off phase, typically spans from 40% to 70% of one gait cycle. This phase is crucial not only for propelling the body forward but also for vaulting the center of mass over the stance leg (Dubin, 2014). It serves as the primary phase of mechanical work output from the ankle (Dubin, 2014), and the optimal phase for AFEs to support (Zhang et al., 2017). Notably, higher speeds exhibit a greater rotation angle, resulting in increased mechanical work output. Speed also influences kinetics, as evidenced by the average biological torque depicted in Figures 4C, D. As the speed increases, the peak torque increases from approximately 1.1–1.7 Nm/kg, accompanied by an earlier peak time from approximately 50%–45% of the gait cycle. These changes reflect the dynamic nature of biomechanical demands as walking speed varies, emphasizing the importance of adaptable assistance strategies to optimize performance and reduce MC efficiently.

### 3.2 Insight into ankle assistance strategies

**FIGURE 5:**
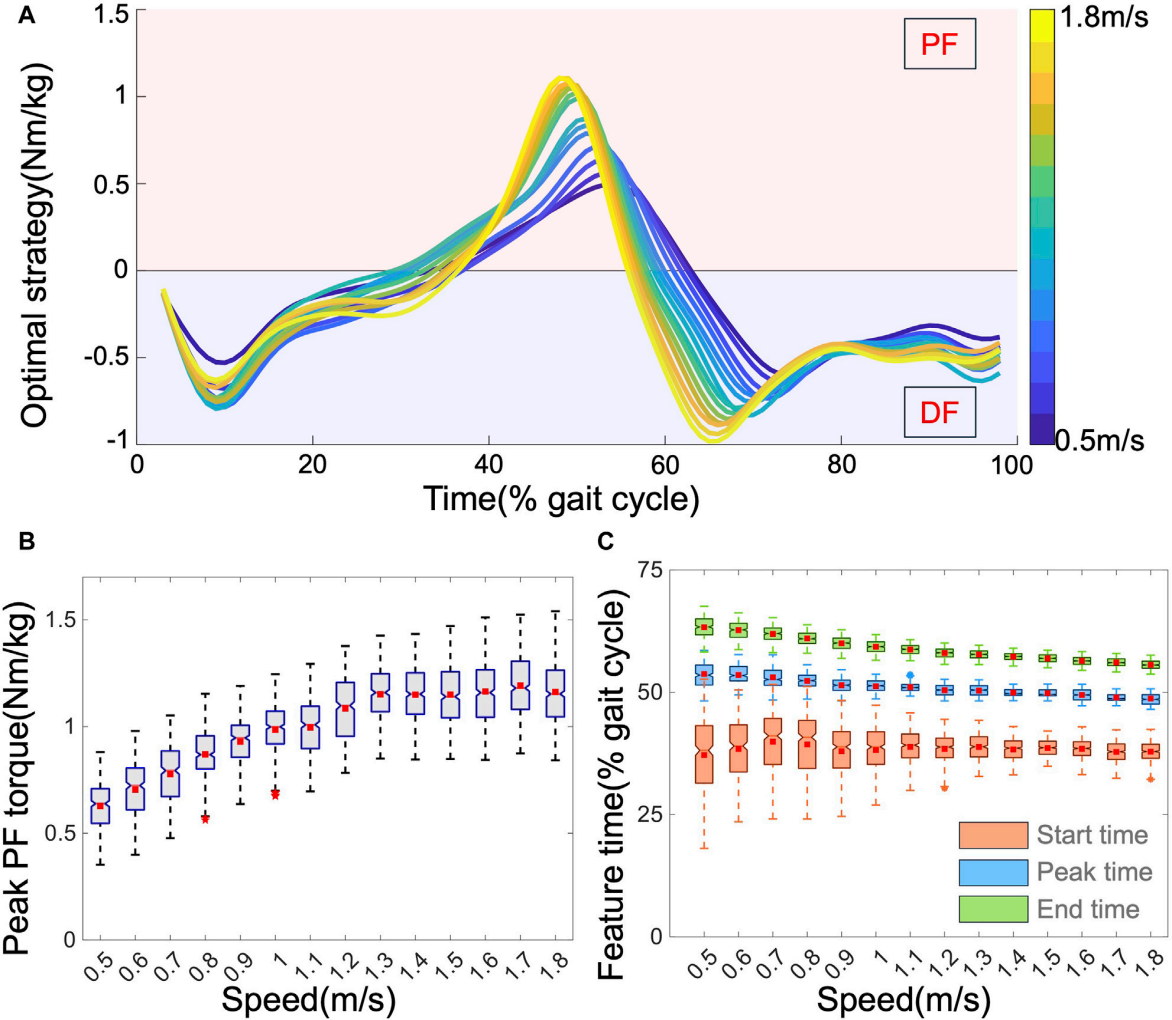
**(A)**. The average OA profiles, normalized by mass. The influence of speed on four features: **(B)** peak PF torque, and **(C)** peak time (blue), start time (orange), and end time (green).

Through MS simulations, we derived the output torque of the AFE called the OA strategy. Figure 5A displays the average OA strategies across all subjects, exhibiting variations in magnitude and timing with speed. Unconstrained in the simulations, the OA strategy supported both PF and DF. PF assistance was active from mid-stance (approximately 40% of the gait cycle) to toe-off (approximately 70% of the gait cycle), coinciding with the propulsion phase of walking. Figures 5B, C depict how the four profile features of the OA strategy varied with speed. Speed impacts these features diversely; as the speed increases, the peak PF torque increases from approximately 0.6–1.2 Nm/kg, with both the peak time and end time advancing (from approximately 55%–48%, and from 65% to 55%, respectively). These observations align with expectations: higher speeds necessitate greater support from AFEs. Moreover, with faster speeds, the leg transitions from the stance to the swing phase more rapidly, resulting in earlier peak and end times. Interestingly, the start time of the OA strategy exhibited less apparent variation with speed and generally fluctuated around 38% of the gait cycle within a more concentrated range. This suggests that the initiation of assistance is relatively stable and hard to predict across different speeds. In addition to speed, other movement characteristics also affected the OA features. For example, both stride length and heel clearance exhibited a strong negative correlation with timing features but a positive correlation with peak torque. Body dimensions, especially the lengths of the thigh and calf, demonstrated a stronger correlation with OA features than with other body features. Comparing the OA strategy with the biological torque profiles revealed notable differences in both magnitude and timing. This suggests that directly mimicking the biological torque profile may not be the optimal choice for exoskeletons.

### 3.3 Evaluation results of the feedforward neural network model

In evaluating the predictive accuracy of our FNN, we focused on four key profile features: peak value, peak time, start time, and end time. This evaluation was conducted both before and after the implementation of transfer learning. We compared the predicted values against the ground truth from simulations. The R-squared metric served as our evaluation criterion, with values closer to 1 indicating higher accuracy. The results, detailed in Table 1, revealed a notable enhancement in prediction accuracy following the application of transfer learning. Initially, while the FNN was proficiently trained on data from 20 subjects, its accuracy was somewhat limited when predicting for new individuals, primarily due to the variability in individual characteristics and the model’s lack of subject-specific tuning. Transfer learning effectively addressed these limitations by leveraging additional data to enhance the model’s ability to new individuals and movement patterns. Consistent with our observed OA strategy, the FNN demonstrated high accuracy in predicting the peak value, peak time, and end time of the profiles. However, the accuracy for predicting the start time was comparatively lower. To further quantify the shape similarity between the profiles generated from ground truth, predicted values, and those obtained from simulations, we employed point-wise profile Euclidean distance and the Chamfer distance as evaluation metrics (both L2 distances, with lower values indicating greater similarity between profiles). Detailed computations based on the test dataset underscored the efficacy of transfer learning in enhancing the congruence between predicted profiles and those generated from ground truth and simulations, as illustrated in Table 2. These evaluations not only demonstrate the robustness and flexibility of our FNN approach but also highlight its potential for real-world applications.

**TABLE 1:** Comparison of prediction accuracy before and after transfer learning (R-squared).

	Peak value	Peak time	Start time	End time
Before	0.6861	0.5912	0.6241	0.6386
After	0.8562	0.7658	0.6849	0.8224

**TABLE 2:** Comparison of shape similarity before and after transfer learning.

	Point-wise profile distance		Chamfer distance	
	Before	After	Before	After
Simulated VS Ground truth	0.1001	0.1001	0.0661	0.0661
Simulated VS Predicted	0.1013	0.0942	0.0675	0.0638
Ground truth VS Predicted	0.0297	0.0232	0.0161	0.0137

### 3.4 Comparing metabolic reductions among assistance strategies

**FIGURE 6:**
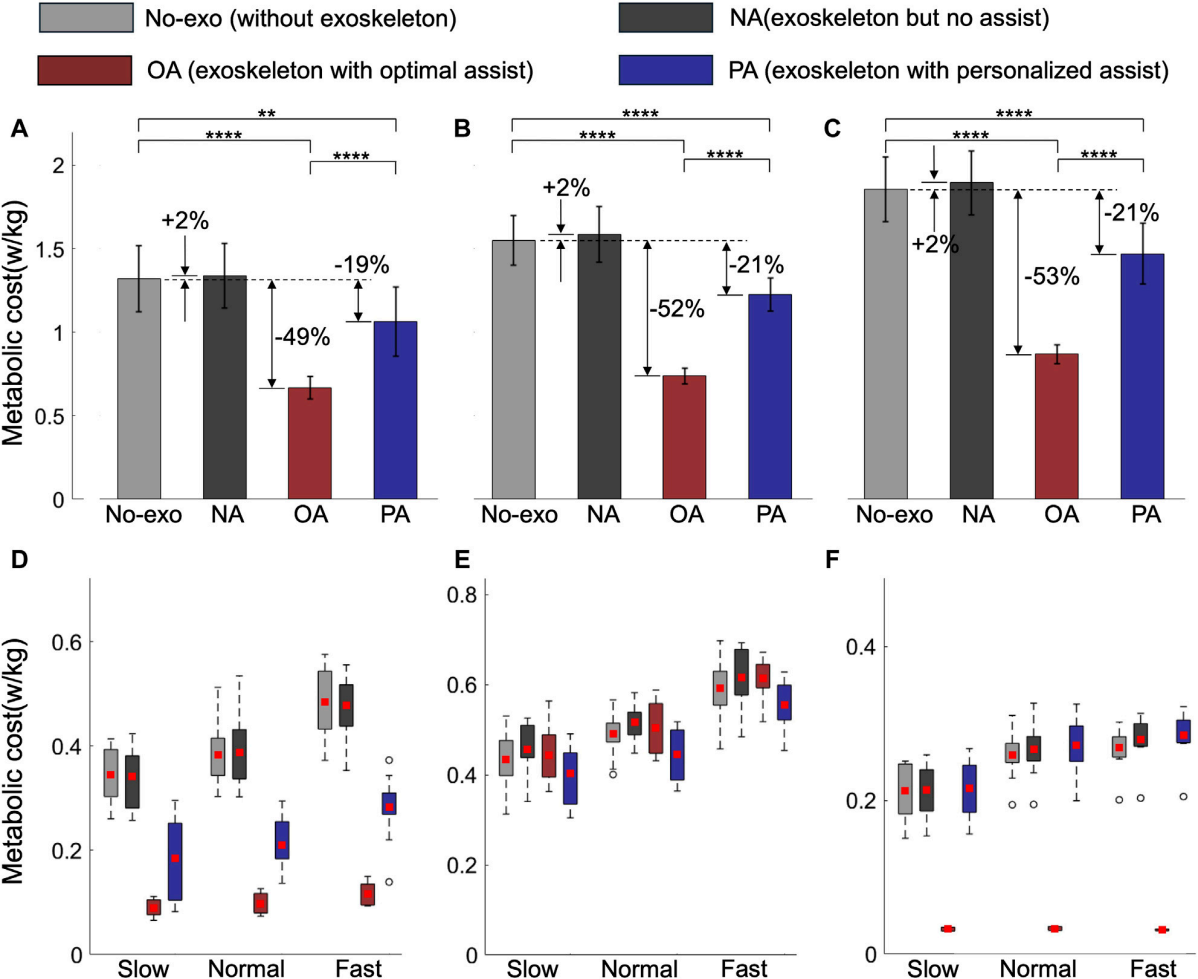
The total calf muscles **(A–C)** and specific muscles **(D–F)** MC under various assistance conditions. The gray, black, red, and blue bars represent the No-exo, NA, OA, and PA conditions, respectively. The percentage change compared to that of the No-exo group is marked next to each bar. The MC values are averaged across two subjects in five gait cycles for each speed and normalized by subject mass. Asterisks denote statistical significance (Tukey post-hoc test, n = 10, **P < 0.01, ****P < 0.0001).

Using MS simulation, we evaluated the MC under various assistance conditions: No-exo (without wearing the AFE), NA (no assistance, wearing the AFE but without assistance), OA, and PA. Given the absence of upper limb muscles, our analysis focused primarily on unilateral calf muscles which govern right ankle joint movement. Figure 6A–C illustrates the average results from five gait cycles per test subject at each speed. We utilized two-way analysis of variance to identify differences. Regardless of assistance, the total MC of calf muscles increased with speed, with the NA condition exhibiting the highest MC (+2%) compared to the others. This observation aligns with prior research (Seethapathi and Srinivasan, 2015), indicating that the MC increases with speed, and adding mass to the leg also elevates the MC (Browning et al., 2007). Both OA and PA decreased the MC compared to the No-exo condition. PA reduced the total calf muscle MC by 0.26+0.05 w/kg (slow, mean ± SD), 0.32±0.08 w/kg (normal), and 0.39±0.10 w/kg (fast), a relative decrease of approximately 20% compared to that of No-exo. Different types of assistance had distinct impacts on specific muscles. We analyzed the MC of the muscles, including the soleus, gastrocnemius, and tibialis anterior, as shown in Figures 6D–F. Under OA conditions, the MC of the tibialis anterior and soleus decreased by approximately 85% and 75%, respectively. However, there was no discernible reduction in the gastrocnemius. Conversely, the PA condition reduced the MC of the soleus and gastrocnemius by approximately 45% and 10%, respectively, while slightly increasing that of the tibialis anterior by 5%.

## 4 Discussion

Motivated by the promising potential of exoskeletons and advancements in MS research, this study explored a more efficient method for personalizing exoskeleton assistance. Through MS analysis, we studied the walking activities of 22 individuals across various movements to identify corresponding OA strategies. These OA strategies notably reduced the total MC of calf muscles by 0.65±0.17 w/kg (slow), 0.81±0.14 w/kg (normal), and 0.99±0.15 w/kg (fast), achieving an approximately 50% reduction compared to normal walking. This finding aligns with previous simulation studies, such as that reported by Dembia et al. (2017), which showed reductions of 0.65±0.28 w/kg and 0.38±0.10 w/kg for PF and DF assistance, respectively. To overcome inherent limitations of MS simulation and develop a more efficient approach, we employed an FNN to establish a mapping from body characteristics and movement features to assistance profiles. This FNN simplifies the process of generating PA strategies.

Our method demonstrates remarkable efficiency in terms of time. While traditional MS simulations typically require several minutes (approximately 8 min per cycle on an Intel-13900 K platform) to complete a single gait cycle, our method delivers PA strategies in milliseconds. Furthermore, our method exhibits greater adaptability and extendibility compared to existing methods, which are often restricted to specific individuals and movements. Our approach obtains PA strategies that not only effectively reduce MC but also swiftly adapt to new individuals and movements. Although this study primarily focused on the ankle joint, the methodology can be expanded to other joints such as the hip and knee by adjusting the parameterization of PA strategies. As outlined by Bryan et al. (2021), suitable parameterizations of PA strategies for different joints can always be identified.

Our PA strategy achieved an approximately 20% reduction in the total MC of calf muscles, indicting a notable improvement in walking economy despite not achieving the 50% reduction attained by the OA strategy. Several factors account for this disparity in effectiveness. Primarily, the OA strategy represents the theoretical pinnacle of assistance efficiency, and any deviation from this optimal configuration is inevitably less efficient. Additionally, while deriving the OA strategy, we did not impose any constraints on its profile, resulting in a highly intricate and impractical design. In contrast, the PA strategy is more practical, especially since most AFEs only assist in PF. It is a predicted outcome derived from parameterizing the OA strategy specifically for PF, making it suboptimal even in the PF direction.

**FIGURE 7:**
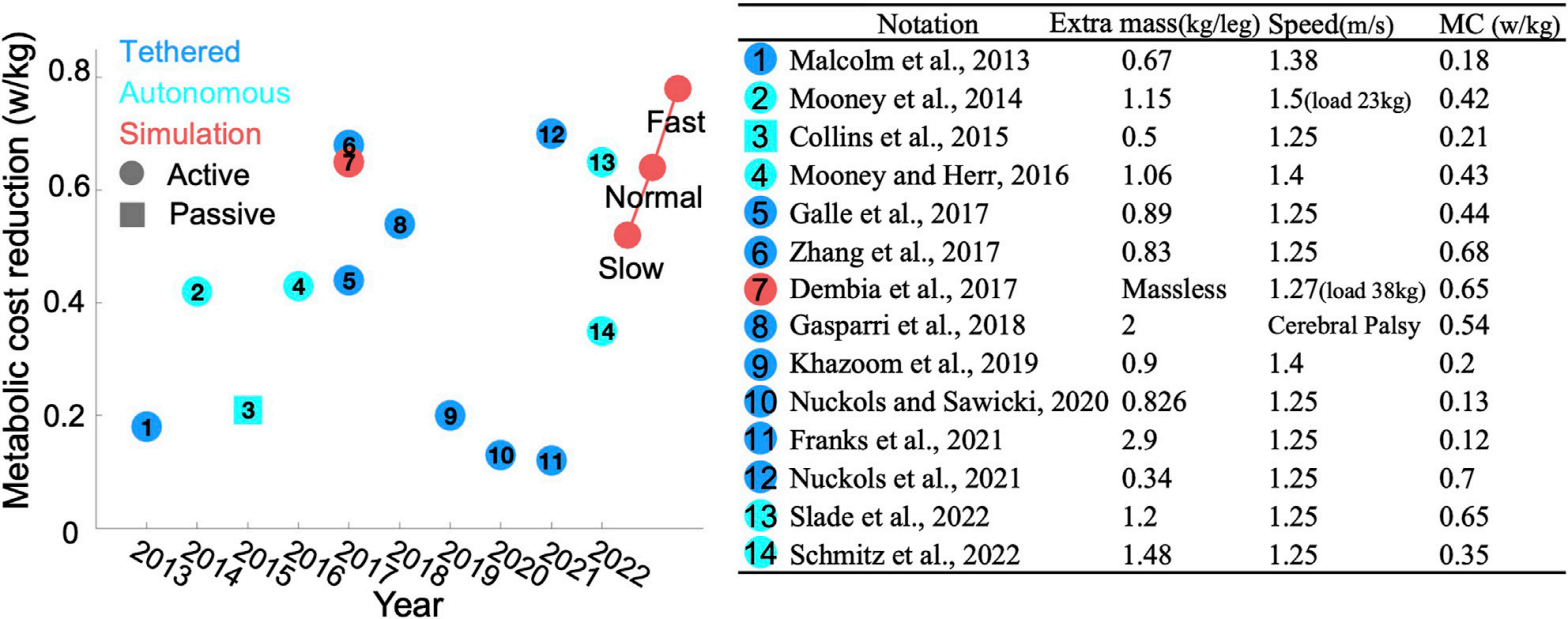
Comparison results: (left) Publishing year of each study versus reduction in MC. The colors blue, cyan, and red represent tethered devices, autonomous devices, and simulations, respectively. Circles denote active devices, while squares denote passive devices. Red circles on the right side highlight our bilateral results. (right) Detailed information for each study referenced on the left.

We compared the efficiency of our PA strategy with that of relevant studies focusing solely on AFEs supporting PF over the past decade (Malcolm et al., 2013; Mooney et al., 2014; Collins et al., 2015; Mooney and Herr, 2016; Dembia et al., 2017; Galle et al., 2017; Zhang et al., 2017; Gasparri et al., 2018; Khazoom et al., 2019; Nuckols and Sawicki, 2020; Franks et al., 2021; Nuckols et al., 2021; Schmitz et al., 2022; Slade et al., 2022), as illustrated in Figure 7. Notably, some studies reported impressive results by subtracting the basal MC before comparison, while others did not, resulting in less appealing outcomes. To ensure fairness, we converted all reductions to absolute values. It should also be noted that experimental results often do not perform as well as simulations. This discrepancy arises primarily from the simplification in simulations, such as neglecting additional mass or oversimplifying human-robot interactions. Additionally, simulations are conducted under ideal conditions, whereas experiments face external disturbances like minor terrain changes, which afect overall performance.

Unlike previous studies that modeled exoskeletons as massless entities (Uchida et al., 2016; Dembia et al., 2017), we considered the additional mass associated with AFEs to enhance the realism of human-robot interactions. Our review of existing AFE designs revealed that AFEs typically comprise two components: one attached to the calf and another to the foot, with a combined mass generally not exceeding 1.5 kg, as depicted in Figure 7. Moreover, commercially available AFEs, such as the ExoBoot, weight approximately 1.9 kg. To maintain generalizability, we modeled the AFE in the same two parts, adhering to a mass ratio of 3:1 and setting the total mass at 2 kg. Additionally, we explored the impact of the AFE mass on the OA strategy, by adjusting the total mass from 1 to 4 kg while maintaining the same ratio. We observed that for every 1 kg increase in extra mass, the peak torque increased by approximately 0.01 Nm/kg. Furthermore, in our initial simulations of the interaction forces between the human body and the AFE, we utilized a linear spring model. However, upon evaluation, we observed that varying the stiffness of the spring didn’t impact the outcomes of the OA strategies but increased the simulation time. Consequently, we decided to rigidly connect the AFE to the human body in our formal simulations to expedite the simulation process.

**FIGURE 8:**
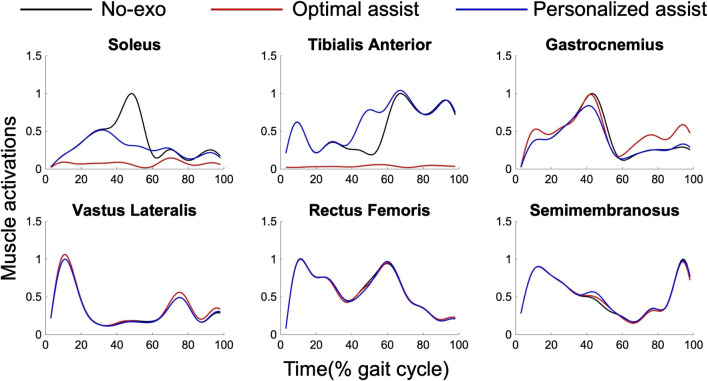
Muscle activations under different assistance conditions. The lines represent the average of the two test subjects walking at a normal speed. The simulation results are filtered to eliminate noise and normalized to the peak value of walking without wearing the exoskeleton (black). The red and blue lines represent the results under the OA and PA conditions, respectively.

This study not only contributes to the personalized design of assistance but also enhances our understanding of human gait biomechanics. We investigated muscle activations under different assistance scenarios, as displayed in Figure 8. Reductions in activations and the MC primarily occurred in muscles that cross the assisted joint and act in the same direction as the assistance. The OA strategy notably reduced activation in the soleus and tibialis anterior, while the effect on the gastrocnemius was less pronounced. This difference may be attributed to the gastrocnemius being a biarticular muscle and receiving lower priority in the CMC algorithm. The PA strategy showed a beneficial effect on both the soleus and gastrocnemius, whereas the tibialis anterior required increased activation to counterbalance the external torque, likely due to muscle antagonism. Additionally, assisting the ankle joint had minimal influence on muscles not directly acting on the ankle joint, such as the vastus lateralis, rectus femoris, and semimembranosus.

However, this study has several limitations. A primary constraint is the assumption that the assistance provided by the AFE does not alter human movements. Contrary to this assumption, some studies (Nuckols et al., 2021; Slade et al., 2022) have indicated that such assistance can increase walking speed, suggesting that dynamic human-robot interactions were not fully captured in our study. Additionally, all the quantitative analyses were based on simulations, which may overestimate the benefits of the AFE and lack practical validation. To address this limitation, we are in the planning stages of conducting human trials with our developed Bowden cable-driven ankle exoskeleton to further validate our method. More accurate and realistic assessments could be achieved through experiments, which would provide more compelling evidence. Other limitations include the relatively small number of individuals used to train the FNN. We used 20 subjects with 6,000 samples, which may insufficiently represent the wider population diversity, and we will add walking data from more subjects in the future.

## 5 Conclusion

This paper explores the potential of PA strategies for enhancing walking economy and proposes a hybrid data-driven approach that integrates MS simulation with ML technology to customize effective and feasible assistance strategies for different users and movement scenarios. Compared to traditional methods, our approach effectively reduces the MC of calf muscle by approximately 20% with lower time consumption and better adaptability. In the future, further empirical research is needed to validate the actual effectiveness of our method. We hope that this study can promote some consensus on PA strategies and provide valuable insights for exoskeleton design.

## Data Availability

The raw data supporting the conclusions of this article will be made available by the authors, without undue reservation.
